# Synthesis, structural characterization, spectroscopic analyses, DFT-modeling, and RDG-NCI assessment of 4-dimethylaminopyridinium dihydrogen monophosphate

**DOI:** 10.3389/fchem.2025.1701702

**Published:** 2025-11-17

**Authors:** Lelfia Guelmami, Mondher Dhifet, Khadija Zaki, Norah Alwadai, Hammadi Khmissi, Mohamed Bouzidi, Bouzid Gassoumi, Mohammed Bouachrine

**Affiliations:** 1 Laboratory of Functional Physiology and Valorization of Bio-Resources (UR17ES27) at the Higher Institute of Biotechnology of Beja (ISBB), University of Jendouba, Jandouba, Tunisia; 2 National Institute of Technology and Sciences of Kef, University of Jendouba, Jandouba, Tunisia; 3 Laboratory of Physico-Chemistry of Materials (LR01ES19), Faculty of Sciences of Monastir, Monastir, Tunisia; 4 Faculty of Sciences of Gafsa, University of Gafsa, Gafsa, Tunisia; 5 Laboratory of Molecular Chemistry and Natural Substances, University of Moulay Ismail, Faculty of Sciences, Meknes, Morocco; 6 Department of Physics, College of Science, Princess Nourah bint Abdulrahman University, Riyadh, Saudi Arabia; 7 Department of Physics, College of Science, Northern Border University, Arar, Saudi Arabia; 8 Department of Physics, College of Science, University of Ha’il, Ha’il, Saudi Arabia; 9 Laboratory of Advanced Materials and Interfaces (LIMA), University of Monastir, Faculty of Sciences of Monastir, Monastir, Tunisia

**Keywords:** dimethylaminopyridinium dihydrogenmonophosphate, IR spectroscopy, DFT calculations, Hirshfeld surfaces analysis, molecular electrostatic potential (MEP) calculation, electron localization function (ELF), localized orbital locator (LOL) analysis

## Abstract

An organoammonium-dihydrogenphosphate compound (C_7_H_11_N_2_)H_2_PO_4_ was synthesized and characterized. The intra-and intermolecular interactions responsible for the stability of our compound within the crystal lattice have been thoroughly discussed. FT-IR spectroscopic analyses have confirmed the well atomic organization and stability of our compound. Using the RDG and NCI approaches, we identified strong N—H···O and O—H···H hydrogen bonds, along with notable van der Waals (vdW) interactions between the cationic units and the phosphate anion, confirming the key role of non-covalent forces in stabilizing the crystal structure. Intermolecular interactions were further elucidated by Hirshfeld surface analysis. Moreover, dispersion-corrected Density Functional Theory provided insights into chemical reactivity properties. The compound was also analyzed using solid-state spectroscopies. This contribution enhances the understanding of the structural diversity of organic-dihydrogenphosphate compounds.

## 1 Introduction

Organophosphorus compounds are important in everyday applications ranging from agriculture to medicine and are used in flame retardants and other materials (Montchamp, 2014). Although organophosphorus chemistry is known as a mature and specialized area, researchers would like to develop new methods for synthesizing organophosphorus compounds to improve the safety and sustainability of these chemical processes. In the last years, several syntheses of organic-cation monophosphate crystals have been carried out. During the systematic investigation of interaction between monophosphoric acid with organic molecules, numerous structures of monophosphates with organic cations have been described: (C_5_H_6_N_5_)H_2_PO_4_ (Vratislav et al., 1979), (C_2_H_6_NO_2_)H_2_PO_4_ (Averbuch-Pouchot et al., 1988), (C_5_H_7_N_2_)H_2_PO_4_.H_2_O (Bag et al., 1991), (C_7_H_10_NO)H_2_PO_4_ (Eaoud et al., 1999), (C_6_H_22_N_4_) (HPO_4_)_2_.2H_2_O (Kamoun et al., 1991), (C_3_H_12_N_2_)HPO_4_.H_2_O (Kamoun and Jouini, 1990), (C_4_H_14_N_2_)HPO_4_.2H_2_O (Kamoun et al., 1990) and (C_4_H_15_N_3_)HPO_4_.2H_2_O (Kamoun et al., 1995), etc. These complexes have been extensively investigated because of their numerous practical and potential uses in various fields such as biomolecular sciences, catalysts, fuel cell, liquid crystal-material developers and quadratic non-linear optic (Gani and Wilkic, 1955; Mass et al., 1993; Olivier et al., 1995; Oueslati et al., 2005; Wang et al., 1996; Yuichi et al., 1995). The phosphate anions generally observed are the acidic ones [HPO4]^2-^ or [H_2_PO_4_]^-^. Such anions are interconnected by strong hydrogen bonds so as to build infinite networks with various geometries: ribbons (Baouab and Jouini, 1998), chains (Averbuch-pouchot and Durif, 1987; Averbuch-Pouchot et al., 1988), two-dimensional networks (Averbuch-Pouchot et al., 1988; Bagieu-Beucher, 1990; Blessing, 1986) and three-dimensional networks (Averbuch-pouchot et al., 1989).

Molecular crystals are crystalline solids consisting of two or more different molecular entities arranged in a periodic lattice structure and held together by intermolecular non-covalent interactions. The combination of organic amines with inorganic dihydrogenphosphate moieties has garnered significant interest due to the possibility of merging the structural and physicochemical properties of both components, leading to the formation of molecular crystals and salts with particular features (Balamurugan et al., 2010; Belghith et al., 2015; Budzikur et al., 2022; Das et al., 2017; Fujita et al., 2009; Guelmami et al., 2023; Kaabi et al., 2003; Mahé et al., 2013; Mrad et al., 2011; Rayes et al., 2016).

In organoammonium dihydrogenphosphates, the structural integrity of the crystals is primarily derived from a supramolecular hydrogen bonding network, particularly O—H···O and N—H···O hydrogen bonds. In addition, C–H … O hydrogen bonds have been observed, which play a supportive role in determining the crystal packing and overall stability (Fujita et al., 2009; Pandian et al., 2015).

In this context, we report herein the chemical preparation of an organic phosphate material, templated with a 4-dimethylaminopyridine derivative (DMAP). The latter molecule is a cyclic diamine, also called N, N-dimethylpyridin-4-amine (IUPAC name), with the chemical formula (CH_3_)_2_NC_5_H_4_N (Balachandran et al., 2014; Singh et al., 2007). The crystals of the studied (C_7_H_11_N_2_)H_2_PO_4_ were synthesized by slow evaporation at room temperature, and was characterized by using single-crystal X-ray diffraction and infrared spectroscopy IR.

To gain deeper insight into the structural, electronic, and intermolecular interaction profile of (C_7_H_11_N_2_)H_2_PO_4_, a comprehensive theoretical investigation was carried out. Density Functional Theory (DFT) calculations were performed to analyze the frontier molecular orbitals (HOMO and LUMO), providing essential information on the compound’s electronic reactivity and kinetic stability. The molecular electrostatic potential (MEP) surface was generated to visualize charge distribution and identify potential sites for nucleophilic and electrophilic interactions. Furthermore, the Electron Localization Function (ELF) and Localized Orbital Locator (LOL) analyses were employed to map electron density localization, revealing bonding characteristics and the presence of lone pairs. The NCI and RDG approaches revealed the presence of significant N—H···O and O—H···H hydrogen bonds, as well as dispersive van der Waals contacts between the organic cations and the phosphate anion, highlighting the key role of non-covalent interactions in the molecular assembly. To complement the theoretical approach, Hirshfeld surface analysis was conducted to quantify and visualize the intermolecular interactions within the crystal packing, offering detailed insight into the nature and extent of hydrogen bonding, π–π stacking, and van der Waals forces that stabilize the solid-state structure.

## 2 Experimental

### 2.1 Chemical preparation

An aqueous solution of monophosphoric acid (85%, d = 1.7) was added to an organic molecule, 4-dimethylaminopyridine, in the molar ratio 1:1. The resulting solution was slowly evaporated at room temperature for 3 weeks until colorless, parallelepipedic crystals of (C_7_H_11_N_2_)H_2_PO_4_ were formed. The structure was determined using single-crystal X-ray diffraction.

The 4-dimethylaminopyridinium dihydrogenmonophosphate (C_7_H_11_N_2_)H_2_PO_4_ denoted as 4-DMAPMP crystallizes in the triclinic system, with the space group P $\overset{–}{1}$ and the following parameters: a = 7.8046(3) Å, b = 8.0826(3) Å, c = 8.4262(3) Å, α = 98.497(3), β = 104.689(3), γ = 99.428(3), V = 497.14(3)Å^3^, Z = 2 (Guelmami et al., 2012). The crystal structure was solved using the direct methods with the program SHELX-97 (S and heldrick, 2008) from the WinGX package (Farrugia, 1997). All non-hydrogen atoms were first refined with isotropic and then with anisotropic displacement parameters. The final cycle of the refinement, including 127 parameters, leads to the reliability factors R_1_ = 3.31% and R_w_ = 8.39%. The average value density D_m_ = 1.452 g cm^-3^, measured at room temperature using toluene as picnometric liquid, is in agreement with the calculated D_c_ = 1.471 g cm^-3^.

### 2.2 Infrared spectroscopy

IR spectrum was recorded at room temperature with a Biored FTS 6000 (F.T.I.R) spectrometer over the wave number range of 4,000–400 cm^-1^ with a resolution of about 4 cm^-1^. Thin transparent pellets were used by compacting an intimate mixture obtained by shaking 2 mg of the sample in 100 mg of KBr.

### 2.3 Computational details–DFT calculations

All quantum chemical calculations were performed using the Gaussian 09 software package (Frisch et al., 2009). The molecular geometry of (C_7_H_11_N_2_)H_2_PO_4_ was fully optimized in the gas phase using the Density Functional Theory (DFT) method with the B3LYP functional and the 6-31 G(d,p) basis set. Frequency calculations were conducted to ensure that the optimized structure corresponds to a true energy minimum, with no imaginary frequencies observed. The B3LYP functional combined with the 6-31G(d,p) basis set was chosen as it offers a well-established balance between accuracy and computational cost and has been widely employed in similar studies of organic and bioactive molecules, ensuring both reliability of the results and comparability with previous work.

### 2.4 Frontier molecular orbital (HOMO–LUMO) analysis

The energies and spatial distributions of the Highest Occupied Molecular Orbital (HOMO) and Lowest Unoccupied Molecular Orbital (LUMO) were extracted from the optimized geometry. The HOMO–LUMO energy gap (ΔE) was calculated to assess the kinetic stability and electronic reactivity of the compound. Orbital visualizations were rendered using GaussView 6.0 (Dennington and Millam, 2000). The Density of States (DOS) was plotted using the GaussSum program (K Boyle et al., 2005).

### 2.5 Molecular electrostatic potential (MEP) mapping

The molecular electrostatic potential surface was computed at the same level of theory (B3LYP/6-31 G(d,p)) based on the optimized structure. The MEP was mapped onto the electron density surface (isovalue = 0.001 a.u.) to visualize charge distribution and predict electrophilic and nucleophilic regions. Color-coded surface plots were generated with red, green, and blue regions representing areas of negative, neutral, and positive potential, respectively (Gassoumi et al., 2023).

### 2.6 Electron localization function (ELF) and localized orbital locator (LOL)

ELF and LOL analyses were performed using the Multiwfn program based on the wavefunction file obtained from Gaussian. These topological functions were computed to assess the localization of electron density within covalent bonds and lone pair regions. 2D and 3D visualizations of the ELF and LOL surfaces were produced to identify zones of high electron localization, particularly around bonding regions, heteroatoms, and delocalized systems (Jacobsen, 2008; Lu and Chen, 2012; Zaki et al., 2024).

### 2.7 RDG and NCI analysis

The optimized geometries were employed for the analysis of non-covalent interactions (NCIs) using the Reduced Density Gradient (RDG) method. Wavefunction files (wfn or.fchk) generated from the DFT-optimized structures were used as input for RDG calculations performed with the Multiwfn 3.8 software package.

The RDG function was evaluated across a three-dimensional grid to map regions of low electron density gradient, which are indicative of non-covalent interactions. The nature and strength of these interactions were further characterized using the sign(λ_2_)ρ descriptor, where ρ denotes the electron density and λ_2_ is the second eigenvalue of the electron density Hessian matrix. This descriptor distinguishes between attractive (negative sign(λ_2_)) and repulsive (positive sign(λ_2_)) interactions, with the density magnitude reflecting interaction strength.

Three-dimensional RDG iso-surfaces were generated and visualized using Visual Molecular Dynamics (VMD), typically with an isovalue of 0.5 a.u. The isosurfaces were color-coded according to the value of sign(λ_2_)ρ: blue regions indicate strong attractive interactions such as hydrogen bonding, green denotes weak dispersive forces like van der Waals interactions, and red highlights repulsive steric effects. Directional arrows were added to facilitate interpretation of interaction sites within the molecular structure.

Additionally, two-dimensional scatter plots of RDG versus sign(λ_2_)ρ were constructed to provide a quantitative analysis of the identified NCIs. These plots help distinguish between different interaction regimes, enabling a clear classification of bonding, dispersive, and steric contributions. The RDG approach, combining topological and visual analysis, thus provided a comprehensive description of the non-covalent landscape within the studied systems (Medimagh et al., 2023).

### 2.8 Hirshfeld surface analysis

Hirshfeld surface analysis was performed using CrystalExplorer 17.5 (Hirshfeld, 1977; Spackman et al., 2021) to quantitatively and visually examine the intermolecular interactions within the crystal structure. The molecular surface was mapped using several descriptors, including the normalized contact distance (d_norm_), shape index, curvedness, internal distance (d_i_), and external distance (d_e_). The d_norm_ mapping was used to highlight regions of close intermolecular contacts, with red, white, and blue areas indicating distances shorter than, equal to, or longer than the sum of van der Waals radii, respectively. The shape index and curvedness surfaces were employed to identify potential π···π stacking interactions and to assess the local surface curvature, respectively. Additionally, fragment patch mapping was conducted to visualize the spatial distribution and identity of adjacent interacting molecules (Spackman and Jayatilaka, 2009; Spackman and McKinnon, 2002).

### 2.9 Fingerprint plot generation

Two-dimensional (2D) fingerprint plots were generated from the Hirshfeld surface data to provide a detailed, quantitative representation of the intermolecular contacts. These plots were constructed by calculating the internal (d_i_) and external (d_e_) distances from each point on the Hirshfeld surface to the nearest atom within the same and neighboring molecules, respectively. The resulting (d_i_, d_e_) pairs were plotted using a color gradient to indicate contact frequency. The overall fingerprint was subsequently decomposed into individual contact contributions (e.g., H···H, N···All, O···H, H···O), allowing for the identification and quantification of each interaction type’s relative contribution to the crystal packing.

## 3 Results and discussion

### 3.1 Structure description

**FIGURE 1:**
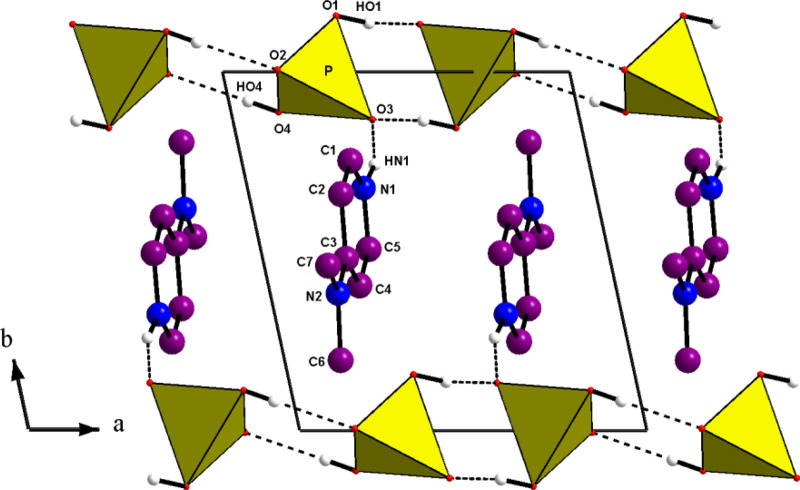
Projection along the c direction of the atomic arrangement of 4-DMAPMP.

**FIGURE 2:**
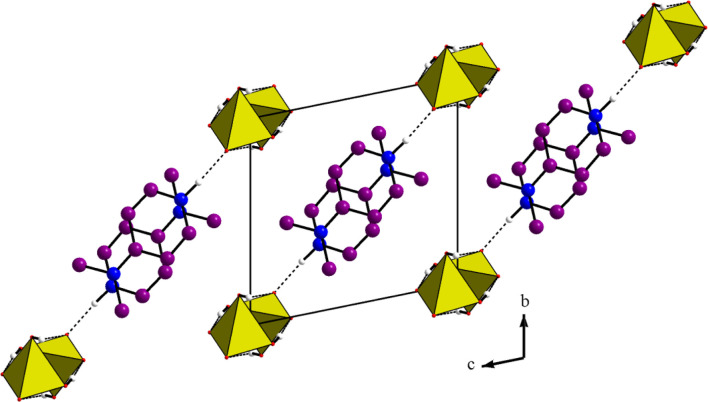
Projection along the a direction of the atomic arrangement of 4-DMAPMP.

The main feature of the atomic arrangement in (C_7_H_11_N_2_)H_2_PO_4_ is the existence of infinite chains with the formula ${\left[H_{2}{\text{PO}}_{4}\right]}_{n}^{n-}$ . Each ${\left[H_{2}{\text{PO}}_{4}\right]}^{-}$ group is connected to its adjacent neighbors by two strong O—H…O, hydrogen bonds, giving rise to infinite chains spreading along the a-direction (see Figure 1). The ${\left[H_{2}{\text{PO}}_{4}\right]}^{-}$ anions in 4-DMAPMP are linked by strong hydrogen bonds, O(1)—H(O1)…O(3) and O(4)—H(O4)…O(2), to form the observed infinite chains. Since the OLO distances involved in this hydrogen bonding scheme [2.569(1)–2.603(1) Å] (Brown, 1976) are in the same order of magnitude as in the H_2_PO_4_ acidic tetrahedron [2.464(2)–2.546(1) Å] (Guelmami et al., 2012), the ${\left[H_{2}{\text{PO}}_{4}\right]}_{n}^{n-}$ infinite chains should be considered as a polyanion. The P–P distance observed in the chain is 4.092(1) Å. Figure 1 seems to show that aminopyridinium cycles, extending nearly along the b-direction, are anchored to successive mineral anions. However, a projection of the atomic arrangement, along the a-direction (see Figure 2) proves that connection, by the hydrogen bonds N—H…O, is made along the [011] crystallographic axis; the organic entities, not connected together, are anchored onto both anionic chains in the (011) planes. This interaction contributes, with the Van der Waals ones, to the cohesion of the structure.

The D(donor)—H…A(acceptor) hydrogen bonds are listed in Table 1 with an upper limit of 1.85(3) Å for the H…A distances and a lower limit of 163.7(1)° for the D—H…A bond angles (Brown, 1976; Steiner and Saenger, 1994). Thus, this structure exhibits two types of hydrogen bonds: O(P)—H…O connects the acidic anions, giving rise to the chains and one N—H…O establishes contact with the anionic chains. This atomic arrangement includes three hydrogen bond donors (1 N and two O atoms) and two hydrogen bond acceptors (two O atoms). The oxygen atom O(2) is single acceptor, whereas O(3) is a twofold acceptor.

**TABLE 1:** Bond lengths (Å) and angles (°) in the Hydrogen-bonding scheme.

​	D—H	H…A	D…A	D—H…A
N(1)—H(N1)…O(3^i^)	1.05(2)	1.65(1)	2.679(1)	163.7(1)
O(1)—H(O1)…O(3^ii^)	0.91(2)	1.71(2)	2.603(1)	164.8(1)
O(4)—H(O4)…O(2^iii^)	0.72(3)	1.85(3)	2.569(1)	170.7(3)

Symmetry codes: (i): x, y+1, z-1; (ii): x+1; -y+2, -z; (iii): x, -y+2, -z.

### 3.2 Infrared spectroscopy

**FIGURE 3:**
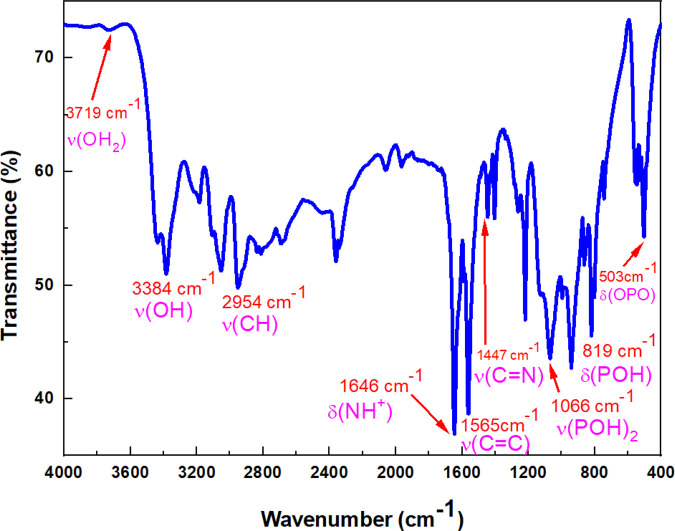
IR spectrum of (C_7_H_11_N_2_)H_2_PO_4_.

The IR spectrum of (C_7_H_11_N_2_)H_2_PO_4_ is depicted in Figure 3. Table 2 describes the spectral data and the band assignments of our compound.

**TABLE 2:** Spectral data and band assignments of (C_7_H_11_N_2_)H_2_PO_4_.

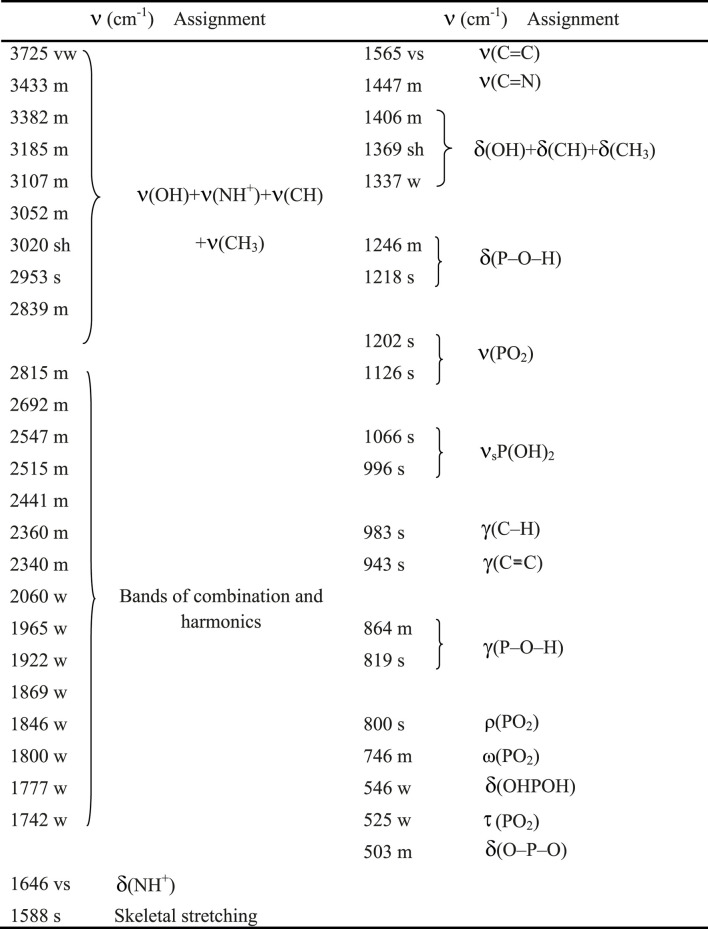

vs., very strong; s, strong; m, medium; w, weak; vw, very weak; sh, shoulder; n, stretching; d, deformation or in-plane bending; g, out-of-plane bending; s, symmetric; as, asymmetric.

The unperturbed PO_4_^3-^ ion is a tetrahedron with point group symmetry *T*_*d*_. The normal modes of vibrations have wave numbers at approximately 938, 420, 1,017 and 567 cm^-1^ for n_1_(*A*1), n_2_(*E*), n_3_(*F*2), and n_4_(*F*2), respectively (Nakamoto, 1986). All of these modes are Raman active, whereas the triply degenerate modes n_3_ and n_4_ are infrared active. The group-theoretical analysis shows that the number of normal modes of PO_4_ tetrahedron is given by the representation $\Gamma_{\mathrm{int}}=A_{1}+E+2F_{2}$ . The localization of two protons on two of the oxygen atoms reduces the ideal symmetry from *T*_*d*_ to *C*_*2v*_. The correlation of group to subgroup shows that these modes can be divided into $2A_{1}+B_{1}+B_{2}$ stretching and bending vibrations $2A_{1}+A_{2}+B_{1}+B_{2}$ in the *C*_*2v*_ symmetry of the H_2_PO_4_ group. Meanwhile, in the crystal, the H_2_PO_4_ has the lower local symmetry *C*_1_, and therefore anisotropic crystal fields may lift degeneracy and allow inactive modes to be active. The interpretation of the IR spectrum (see Figure 3) is made in terms of internal modes of two atomic groups, PO_2_ and P(OH)_2_, included in $H_{2}P{O_{4}}^{-}$ four anions. The two stretching vibrations, asymmetric and symmetric, of PO_2_ group, are observed respectively at 1,150 and 1,074 cm^-1^; while those related to P(OH)_2_ occur as two intense bands, at 940 and 874 cm^-1^ (Haile et al., 1998). Then we attribute the four intense bands at 1,202, 1,126, 1,066, and 966 cm^-1^ to these four vibrations in the 4-DMAPMP compound. The splitting of *F*_2_ stretching mode of PO_4_ into three intense components at 1,202, 1,126, 1,066 cm^-1^ corroborates the symmetry lowering of H_2_PO_4_ in the solid state. On the other hand, bending modes of H_2_PO_4_ group are observed at lower frequencies. The strong, medium and weak bands at 800, 524 and 546 cm^-1^ correspond respectively to the rocking ρ(PO_2_), wagging ω(PO_2_), and to the bending d(OHPOH) vibrations. The two bands, weak at 525 cm^-1^ and medium at 503 cm^-1^, correspond to the torsion τ (PO_2_) and to the bending d(O–P–O) vibrations. Bands at 1,264 and 1,218 cm^-1^ are attributed to in-plane bending d(P–O–H), the out-of-plane bending vibrations g(P–O–H) are observed at 864 and 819 cm^-1^. Frequencies in the range 3,725–2,839 cm^-1^ are attributed to the stretching modes of organic cations and OH groups. d(OH), d(CH) and d(CH_3_) are observed in the range 1,406–1,337 cm^-1^. The presence of the very strong and medium bands at 1,565 and 1,447 cm^-1^ corresponds to the stretching vibration modes of C=C and C=N groups, respectively (Ben Hamada and Jouini, 2006).

### 3.3 Theoretical FT-IR spectrum

**FIGURE 4:**
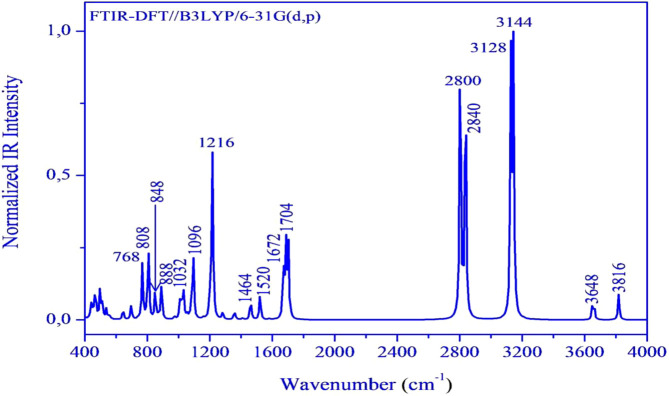
FT-IR spectrum of the (C_7_H_11_N_2_)H_2_PO_4_ calculated at DFT/B3LYP/6-3G(d,p).

Herein, we have meticulously examined the vibrational assignments of the functional groups present in our material. The infrared spectrum of (C_7_H_11_N_2_)H_2_PO_4_ is illustrated in Figure 4. The calculated frequencies have been scaled by a factor of 0.9608 to account for anharmonic effects. Notably, vibrational modes of varying intensities-low, medium, and high-are observed at 3,666 cm^-1^, 3,505 cm^-1^, 3,005 cm^-1^, 2,850 cm^-1^, 2,729 cm^-1^, and 2,690 cm^-1^ corresponding to the stretching vibrations of–OH, –NH, –CH, –CH_3_ groups and harmonics, which corroborate the experimental vibrational frequencies, which are localized at 3,433 cm^-1^ to 3,725 cm^-1^, 2,829 cm^-1^ and 3,020 cm^-1^, 2,815 cm^-1^, 2,692 cm^-1^, respectively. The bending modes observed, ranges from 1,407 cm^-1^ to 1,638 cm^-1^ are attributed to various functional groups including–OH, –CH_3_, –NH, –C=N, –C=C, as well as skeletal stretching vibrations. These frequencies have been experimentally confirmed to fall within the same range. The streaching modes corresponding to the P(OH)_2_ groups are observed at 992 cm^-1^ and 1,053 cm^-1^, confirming experimental findings at 996 cm^-1^ and 1,066 cm^-1^. Furthermore, the frequency values ranging from 738 cm^-1^ to 853 cm^-1^, which were experimentally recorded between 746 cm^-1^ and 864 cm^-1^ are associated with the vibrations of the phosphate group ω(PO_2_), ρ(PO_2_), and g(P–O–H) stretching vibrations. Finally, frequency peaks below 600 cm^-1^, specifically at 484 cm^-1^ and 499 cm^-1^ are linked to the assignments of PO_2_ and O–P–O groups. It is concluded that a strong correlation between the theoretical and experimental results substantiates the robust atomic organization and stability of our compound within the crystal lattice.

### 3.4 Hirshfeld surface analysis

**FIGURE 5:**
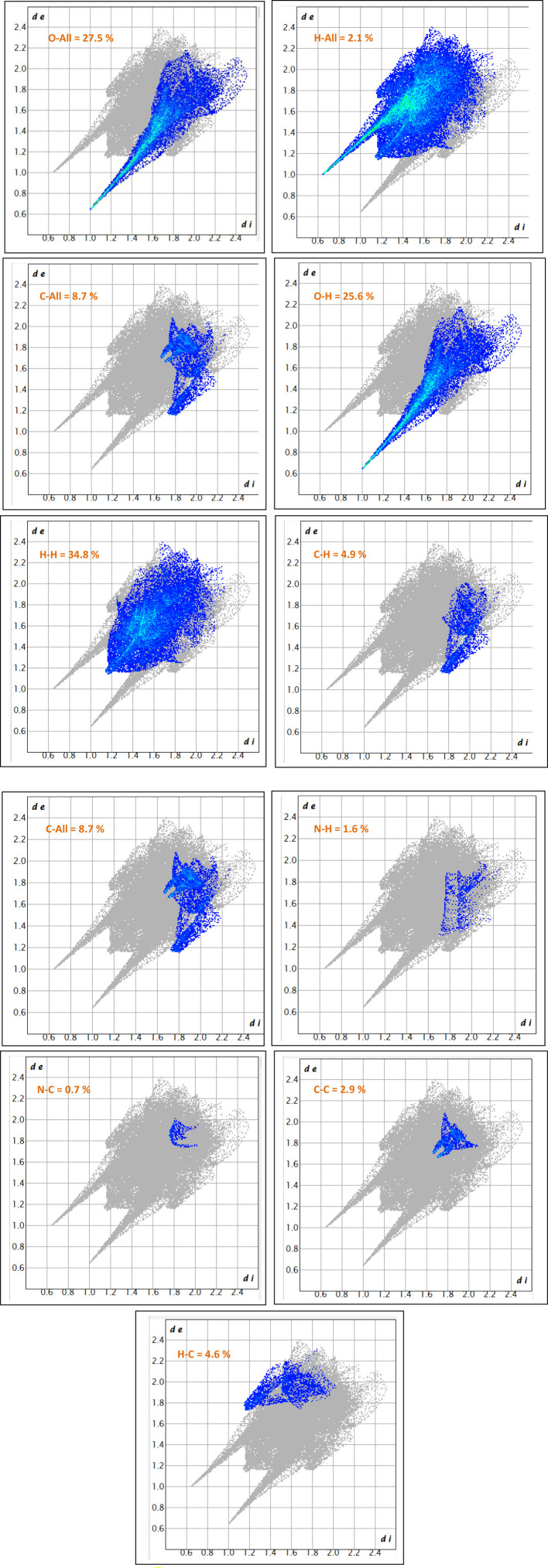
The 2D-fingerprint plot of (C_7_H_11_N_2_)H_2_PO_4_.

**FIGURE 6:**
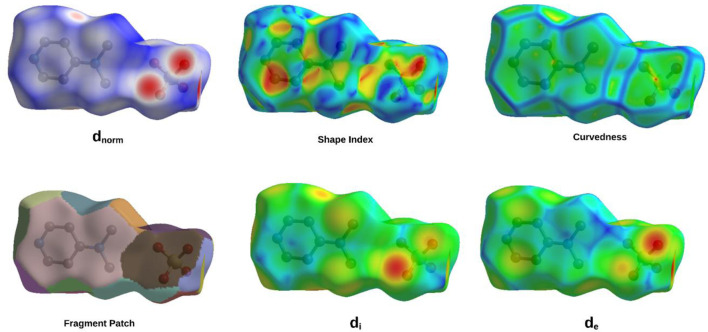
Hirshfeld surface maps of (C_7_H_11_N_2_)H_2_PO_4_ highlighting key intermolecular interactions via D_norm_, D_i_, De, fragment patch, curvedness, and shape index.

The fingerprint plot analysis, displayed in Figures 5, 6, reveals a well-defined hierarchy of intermolecular contacts that collectively orchestrate its crystal packing. Dominating the interaction landscape, N-involved contacts (N–All) account for an exceptional 57.6% of the Hirshfeld surface, with sharp spikes at low *di* and *de* values signifying strong, directional hydrogen bonds—confirming nitrogen’s central role in stabilizing the supramolecular framework. These are robustly complemented by H…H contacts (34.8%), whose broad, diffuse region reflects widespread van der Waals forces, essential for efficient packing and void minimization. Oxygen-mediated interactions, particularly O…H (25.6%) and H…O (21.7%), display distinct low-distance spikes characteristic of classical hydrogen bonding motifs (e.g., O–H…O, C–H…O), cumulatively contributing nearly 47.3%, and underscoring the critical role of oxygen atoms in forming a dense, directional hydrogen-bond network. The broader O–All category (27.5%) reinforces the oxygen atoms’ significance as key interaction sites. Beyond these major contributors, C-involved interactions (C–All: 8.7%, C–H: 5.1%, H–C: 4.6%) and minor contacts involving C–C (3.2%), N–C (2.2%), and O–O (2.1%) offer structural fine-tuning, enabling subtle but meaningful adjustments to the packing efficiency and cohesion. Lesser interactions like N–H (1.6%), O–C (0.6%), and H–All (2.1%) play marginal roles, reflecting either low-frequency occurrences or weaker binding scenarios. Altogether, this quantitative contact mapping firmly establishes nitrogen-mediated hydrogen bonding as the principal force steering the crystal assembly, reinforced by a robust hydrogen-bond network involving oxygen and a supportive matrix of van der Waals and dispersive contacts. Such detailed insight into atomic-level interactions provides a solid basis for understanding the compound’s stability, packing behavior, and potential functional properties in the solid state.

The comprehensive Hirshfeld surface analysis of (C_7_H_11_N_2_)H_2_PO_4_ (Figure 6), integrating the d_norm_, Shape Index, Curvedness, di, and de surfaces, reveals a cohesive picture of the compound’s intricate intermolecular landscape. The d_norm_ surface clearly highlights pronounced red spots—particularly on the oxygen atoms of the phosphor group—signifying strong, localized contacts such as O–H…O hydrogen bonds, which dominate the crystal cohesion. Complementing this, widespread white areas suggest close packing dominated by non-specific van der Waals interactions, while the diffuse blue regions point to zones of lower contact density or minor voids. The Shape Index further refines this picture, displaying characteristic adjacent red and blue triangular motifs on the aromatic regions, indicative of π…π stacking interactions; this underscores the role of precise shape complementarity in molecular alignment. The curvedness surface adds another dimension, with flat, low-curvature zones (green/yellow) corresponding to extended stacking or planar contacts, and highly curved patches (blue/red) identifying discrete localized interactions at molecular peripheries. Meanwhile, the d_i_ and d_e_ surfaces offer a spatial dissection of internal versus external electron density, with red zones on the d_e_ map—particularly around oxygen atoms—confirming the close proximity of hydrogen donors from neighboring molecules, thus corroborating the hydrogen bonding observed in the d_norm_ map. Altogether, this multi-surface interpretation illustrates how (C_7_H_11_N_2_)H_2_PO_4_ balances strong directional hydrogen bonding, π-stacking, and van der Waals forces to stabilize its crystal lattice. These qualitative observations can be further quantified via two-dimensional fingerprint plots, offering a statistical breakdown of each contact type’s contribution to the overall solid-state architecture.

### 3.5 Electronic properties

**FIGURE 7:**
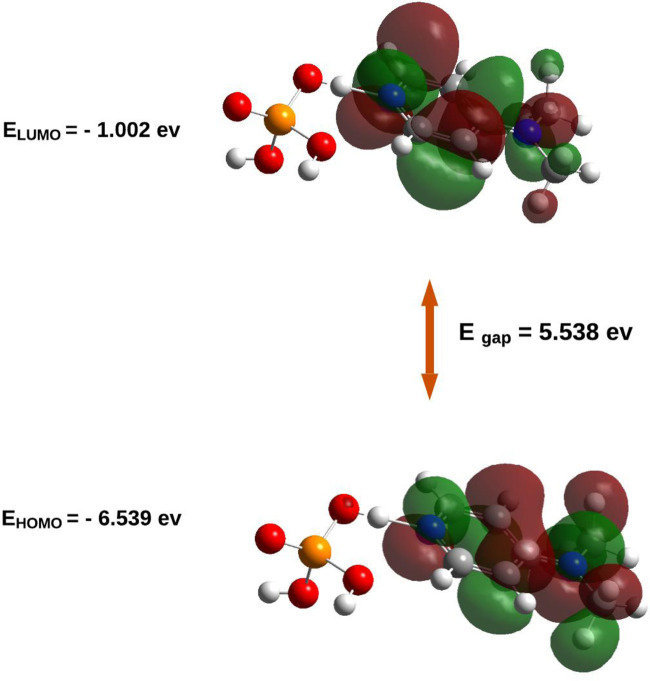
HOMO/LUMO energies and orbital visualization calculated at B3LYP/6-31G(d,p) level of theory.

**FIGURE 8:**
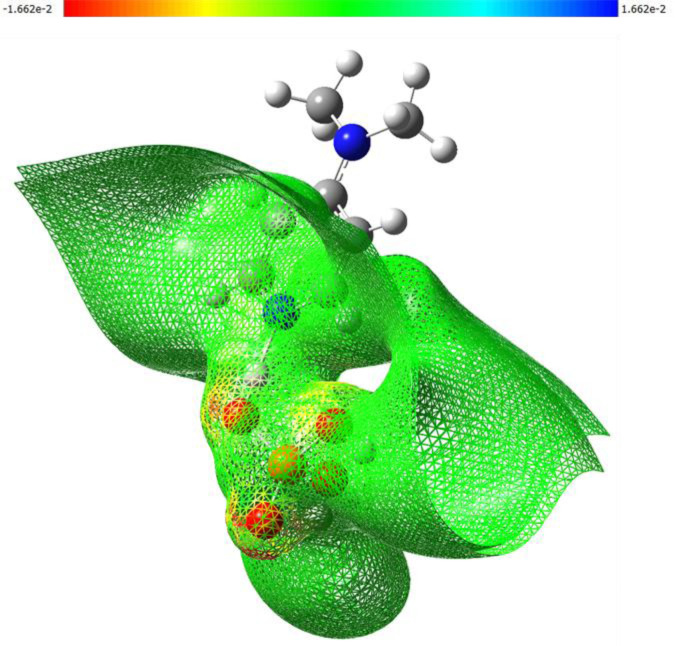
3D-MEP surfaces illustrating the electrostatic potential distribution calculated at B3LYP/6-31G (d,p) level of theory.

The results of this analysis are summarized in Figures 7, 8.

The electronic properties of (C_7_H_11_N_2_)H_2_PO_4_were elucidated through an analysis of its frontier molecular orbitals (Figure 8). The highest occupied molecular orbital (HOMO), with an energy of −6.539 eV, is primarily localized on the organic cation, exhibiting significant electron density around the nitrogen atoms and their adjacent carbon centers. This spatial distribution suggests that these regions are the most accessible for electron donation, influencing the compound’s nucleophilic character. Conversely, the lowest unoccupied molecular orbital (LUMO) was calculated at −1.002 eV, with its electron density also largely concentrated over the organic cation, particularly around the nitrogen atoms and the conjugated ring system. This indicates the primary sites for electron acceptance, defining the compound’s electrophilic nature. The substantial energy gap (E_gap_) between the HOMO and LUMO was determined to be 5.538 eV. This relatively large energy gap is indicative of the compound’s significant kinetic stability, suggesting that considerable energy is required for electronic excitation or charge transfer processes, thereby contributing to its overall chemical inertness under typical reaction conditions.

**FIGURE 9:**
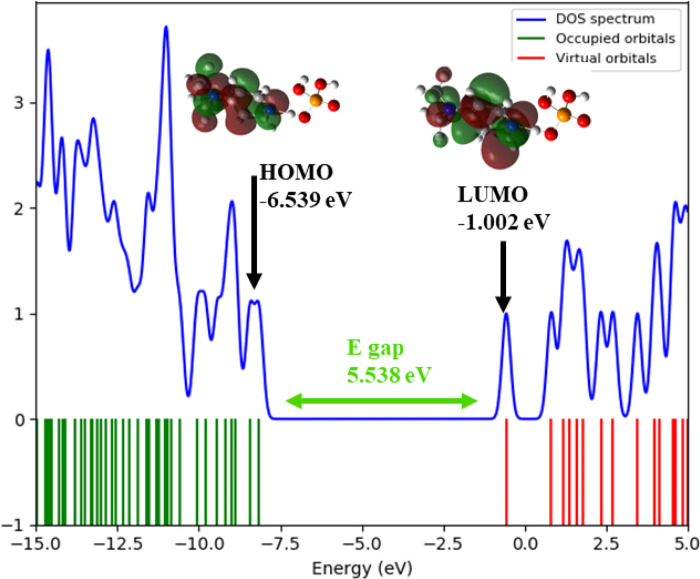
. Density of States (DOS) plot that illustrates the HOMO, LUMO, and energy gap of (C_7_H_11_N_2_)H_2_PO_4_.

This electronic structure is further supported by the Density of States (DOS) spectrum, which offers a more comprehensive view of the distribution of electronic states across the energy range. As depicted in Figure 9, the DOS plot reveals that the electronic states are densely populated below the Fermi level, corresponding to the occupied orbitals (green lines), with a sharp decrease in state density near the HOMO level (−6.54 eV). Above the Fermi level, a clear gap is observed before the reappearance of virtual orbitals (red lines), commencing at the LUMO level (−1.001 eV). The total DOS profile, represented by the blue curve, confirms the absence of states within this 5.537 eV energy window, thus reinforcing the presence of a wide HOMO–LUMO gap. This electronic configuration corroborates the high chemical stability and low reactivity of the compound under ambient conditions, consistent with its insulator-like behavior.

The molecular electrostatic potential (MEP) surface of (C_7_H_11_N_2_)H_2_PO_4_, as depicted in Figure 8, provides a comprehensive visualization of its charge distribution and potential sites for intermolecular interactions. The map clearly identifies regions of pronounced negative potential (red contours, reaching −1.662 × 10^−2^ a.u.) predominantly centered on the oxygen atoms of the dihydrogen phosphate. This high electron density on the phosphate oxygen atoms renders them potent nucleophilic sites and efficient hydrogen bond acceptors, critical for forming stabilizing interactions within the crystal lattice. In contrast, while positive potential regions (blue contours) are less intensely visualized on the organic cation, the overall surface of the organic moiety appears neutral to slightly positive (green to yellow), indicating the delocalization of the positive charge associated with the protonated nitrogen atom. The distinct electrostatic separation between the anionic and cationic components, as visualized by the MEP map, supports the formation of a robust hydrogen-bonding network and highlights the importance of electrostatic forces in the crystal packing arrangement.

### 3.6 Electron localization function (ELF) and localized orbital locator (LOL) analysis

**FIGURE 10:**
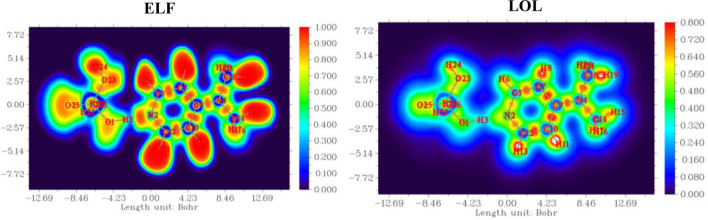
Electron localization function (ELF) and localized orbital locator (LOL) maps of (C_7_H_11_N_2_)H_2_PO_4_.

To gain a comprehensive understanding of the electron localization patterns and bonding characteristics within the complex, both the Electron Localization Function (ELF) and the Localized Orbital Locator (LOL) were computed, as presented in Figure 10.

Both functions consistently revealed prominent regions of high electron localization (red to yellow contours, approaching values of 1.0) around the atomic nuclei, corresponding to the tightly bound core electrons. Critically, these high-localization basins extended between bonded atoms (C-C, C-N, C-H), unequivocally depicting the presence of localized electron pairs forming strong covalent bonds. Furthermore, distinct high-ELF and high-LOL basins were identified on the nitrogen atoms, signifying either localized lone pairs or highly polarized electron density associated with the N-H bonds in the protonated species. Intermediate values (green to cyan) were observed across the heterocyclic ring system, indicative of a degree of electron delocalization that contributes to its overall stability and aromatic character. These complementary analyses, while derived from different theoretical frameworks, consistently underscore the localized nature of core and bonding electrons, providing robust visual evidence for the compound’s intrinsic bonding topology and highlighting specific electron-rich regions that are fundamental to its chemical reactivity and propensity for intermolecular interactions.

### 3.7 NCI-RDG analysis results

**FIGURE 11:**
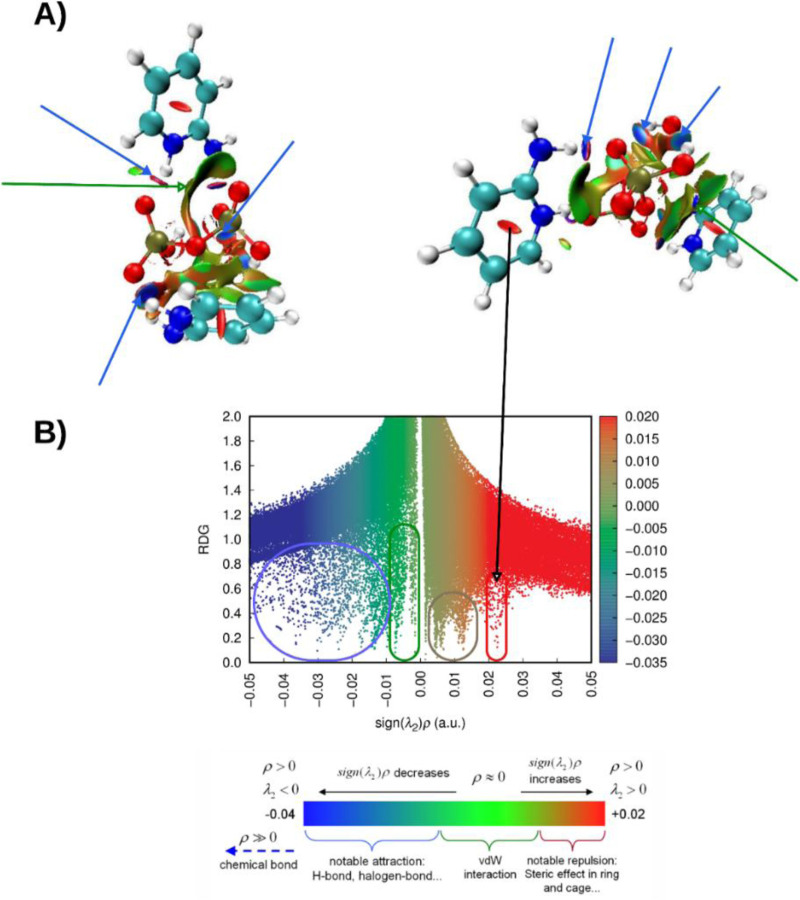
**(A)** NCI analysis of (C_7_H_11_N_2_)H_2_PO_4_ displayed in lateral and axial views, illustrating the spatial distribution of non-covalent interactions. **(B)** Corresponding RDG scatter plot highlighting the interaction regions within the compound (C_7_H_11_N_2_)H_2_PO_4_.

Figure 11 presents a comprehensive analysis of non-covalent interactions (NCIs) within the studied molecular systems, employing the Reduced Density Gradient (RDG) method. This approach provides both visual and quantitative insights into weak intermolecular and intramolecular forces, contributing to a deeper understanding of the electronic factors underlying molecular association, structural stability, and reactivity.

The figure includes a scatter plot of RDG values plotted against sign(λ_2_)ρ, where ρ denotes the electron density and λ_2_ is the second eigenvalue of the electron density Hessian matrix. This representation offers a topological and energetic characterization of the observed interactions. The RDG values, shown on the y-axis, tend toward zero in regions of significant interactions and increase in regions with minimal or no interaction. The x-axis, sign(λ_2_)ρ, distinguishes attractive interactions (negative values) from repulsive ones (positive values), while the electron density magnitude reflects the strength of the interaction. A color gradient overlays the plot to visually reinforce the classification of different interaction types based on their electronic signatures.

The distribution of points reveals three main interaction regimes. On the left side, strong attractive interactions, such as hydrogen bonding and halogen bonding, form a dense cluster characterized by highly negative sign(λ_2_)ρ values and low RDG. The central region, where sign(λ_2_)ρ values approach zero, corresponds to weak dispersive forces, including van der Waals interactions. On the right, positive sign(λ_2_)ρ values and low RDG indicate strong repulsive interactions, typically resulting from steric hindrance.

In this analysis, steric repulsions appeared to occupy a relatively small portion of the scatter plot. In contrast, strong attractive interactions such as hydrogen bonding, specifically NH···O and OH···H contacts, were clearly identified by the prominent green-blue and blue RDG isosurfaces. Additionally, a broad green isosurface, indicative of van der Waals interactions, was observed between the two cationic unit [C_7_​H_11_​N_2_]^+^ and the anion [H_2_PO_4_]^-^. This spatial feature supports previous computational findings and aligns well with the experimental data, reinforcing the structural relevance of these non-covalent contacts.

## 4 Conclusion

In this study, we synthesized a novel organoammonium–dihydrogenphosphate hybrid compound, (C_7_H_11_N_2_)H_2_PO_4_. They were characterized by X-ray diffraction. The results of IR spectroscopy confirm the molecular structure of the studied compound and were found to be in good agreement with X-ray diffraction observations.

The structural and electronic investigations of (C_7_H_11_N_2_)H_2_PO_4_ provide a comprehensive understanding of the factors governing its crystal stability and molecular behavior. Hirshfeld surface analysis revealed that nitrogen-mediated hydrogen bonding plays a central role in the crystal architecture, establishing a directional network that forms the backbone of the supramolecular assembly. Additionally, strong O–H···O interactions involving the phosphate oxygen atoms significantly reinforce lattice cohesion. The presence of π–π stacking and van der Waals forces further contributes to an efficient and orderly molecular packing.

Complementary insights from molecular electrostatic potential (MEP) mapping highlighted distinct zones of negative potential over the phosphate group, identifying key sites for nucleophilic interactions and hydrogen bond formation, while the organic cation exhibited a more delocalized, slightly positive surface. Electron Localization Function (ELF) and Localized Orbital Locator (LOL) analyses confirmed the presence of highly localized electron basins along covalent bonds and lone pairs, particularly around the nitrogen atoms, with moderate delocalization across the aromatic system contributing to overall electronic stability. Using the RDG and NCI approaches, we identified strong NH···O and OH···H hydrogen bonds, along with notable van der Waals interactions between the cationic units and the phosphate anion, confirming the key role of non-covalent forces in stabilizing the crystal structure.

Frontier molecular orbital (FMO) analysis demonstrated that both the HOMO and LUMO are primarily localized on the organic cation, with a significant HOMO–LUMO energy gap (5.538 eV) indicating considerable kinetic stability and limited intrinsic reactivity under ambient conditions.

Collectively, the synergy between directional hydrogen bonding, electrostatic complementarity, covalent bonding topology, and electronic delocalization elucidates the fundamental interactions responsible for the compound’s solid-state integrity and its stable, well-ordered crystal structure.

## Data Availability

The raw data supporting the conclusions of this article will be made available by the authors, without undue reservation.
