# Supplementary material for: Synthesis, structural characterization, spectroscopic analyses, DFT-modeling, and RDG-NCI assessment of 4-dimethylaminopyridinium dihydrogen monophosphate
Source: Front Chem. 2025 Nov 17;13:1701702. doi: 10.3389/fchem.2025.1701702 (PMC12666532; doi:10.3389/fchem.2025.1701702)

checkCIF/PLATON report

Structure factors have been supplied for datablock(s) I

THIS REPORT IS FOR GUIDANCE ONLY. IF USED AS PART OF A REVIEW PROCEDURE FOR PUBLICATION, IT SHOULD NOT REPLACE THE EXPERTISE OF AN EXPERIENCED CRYSTALLOGRAPHIC REFEREE.

No syntax errors found. CIF dictionary Interpreting this report

Datablock: I

```
Bond precision:   C-C = 0.0020 A          Wavelength=0.71073

Cell:           a=7.8046(3)       b=8.0826(3)       c=8.4262(3)
                alpha=98.497(3)   beta=104.689(3)   gamma=99.428(3)
Temperature:    293 K
```

	Calculated	Reported
Volume	497.14(3)	497.14(3)
Space group	P -1	P -1
Hall group	-P 1	-P 1
Moiety formula	C7 H11 N2, H2 O4 P	?
Sum formula	C7 H13 N2 O4 P	C7 H13 N2 O4 P
Mr	220.16	220.16
Dx,g cm-3	1.471	1.471
Z	2	2
Mu (mm-1)	0.269	0.269
F000	232.0	232.0
F000'	232.32	
h,k,lmax	9,10,10	12,13,14
Nref	2167	2133
Tmin,Tmax	0.908,0.948	0.855,0.986
Tmin'	0.898	

```
Correction method= # Reported T Limits: Tmin=0.855 Tmax=0.986
AbsCorr = MULTI-SCAN

Data completeness= 0.984          Theta(max)= 26.987

R(reflections)= 0.0331( 1453)         wR2(reflections)= 0.0839( 2133)

S = 1.109                  Npar= 127
```

The following ALERTS were generated. Each ALERT has the format
test-name_ALERT_alert-type_alert-level.
Click on the hyperlinks for more details of the test.

Alert level C

```
PLAT001_ALERT_1_C No _shelx_res_file DataName Found in SHELXL CIF      Please Do !
PLAT790_ALERT_4_C Centre of Gravity not Within Unit Cell: Resd.  #          1 Note
              C7 H11 N2
PLAT911_ALERT_3_C Missing FCF Refl Between Thmin & STh/L=    0.600         29 Report
                0 -3  1,  -2 -2  1,  -4 -1  1,  -2 -1  1,   0 -1  1,  -1  0  1,
                1  0  1,  -4  3  1,   1  3  1,   4 -3  2,   1 -2  2,  -2 -1  2,
                2 -1  2,  -1  0  2,   2  0  2,   2  1  2,   0  2  2,  -3  4  2,
                1 -4  3,   2 -4  3,   0 -3  3,  -1 -2  3,   1 -2  3,   0 -1  3,
                1 -1  3,   2 -1  3,   0 -2  4,   0 -1  4,   2  1  4,
PLAT918_ALERT_3_C Reflection(s) with I(obs) much Smaller I(calc) .          4 Check
PLAT939_ALERT_3_C Large Value of Not (SHELXL) Weight Optimized S .      41.58 Check
```

Alert level G

```
PLAT007_ALERT_5_G Number of Unrefined Donor-H Atoms .............           3 Report
              H300  H100  H400
PLAT012_ALERT_1_G N.O.K.   _shelx_res_checksum Found in CIF ......     Please Check
PLAT154_ALERT_1_G The s.u.'s on the Cell Angles are Equal ..(Note)      0.003 Degree
PLAT199_ALERT_1_G Reported _cell_measurement_temperature ..... (K)        293 Check
PLAT200_ALERT_1_G Reported   _diffrn_ambient_temperature ..... (K)        293 Check
PLAT380_ALERT_4_G Incorrectly? Oriented X(sp2)-Methyl Moiety .....         C6 Check
PLAT380_ALERT_4_G Incorrectly? Oriented X(sp2)-Methyl Moiety .....         C7 Check
PLAT802_ALERT_4_G CIF Input Record(s) with more than 80 Characters          1 Info
PLAT881_ALERT_1_G No Datum for _diffrn_reflns_av_R_equivalents ...     Please Do !
PLAT883_ALERT_1_G Absent Datum for _atom_sites_solution_primary ..     Please Do !
PLAT910_ALERT_3_G Missing FCF Reflection(s) Below Theta(Min)[Deg]=       2.76 Note
                0  1  0,   0  0  1,
PLAT912_ALERT_4_G Missing # of FCF Reflections Above STh/L=  0.600          3 Note
PLAT933_ALERT_2_G Number of HKL-OMIT Records in Embedded .res File         25 Note
                1 -2  2,   0  2  2,  -1 -2  3,  -2 -1  1,   0 -1  3,   2 -1  2,
                2  1  2,  -2 -2  1,   0 -1  4,   0 -2  4,  -3  4  2,  -7 -6  4,
               -4  3  1,  -4 -1  1,  -2 -1  2,   2 -1  3,   2 -4  3,   4 -3  2,
                2  0  2,   4  0  8,  -9 -2  3,   0 -3  3,   1 -4  3,   0 -3  1,
                1  3  1,
PLAT941_ALERT_3_G Average HKL Measurement Multiplicity ..........         1.0 Low
PLAT950_ALERT_5_G Calculated (ThMax) and CIF-Reported Hmax Differ          -3 Units
PLAT951_ALERT_5_G Calculated (ThMax) and CIF-Reported Kmax Differ          -3 Units
PLAT952_ALERT_5_G Calculated (ThMax) and CIF-Reported Lmax Differ.         -4 Units
PLAT953_ALERT_1_G Reported (CIF) and Actual (FCF) Hmax Differ by .          3 Units
PLAT954_ALERT_1_G Reported (CIF) and Actual (FCF) Kmax Differ by .          3 Units
PLAT955_ALERT_1_G Reported (CIF) and Actual (FCF) Lmax Differ by .          4 Units
PLAT961_ALERT_5_G Dataset Contains no Negative Intensities .......     Please Check
PLAT967_ALERT_5_G Note: Two-Theta Cutoff Value in Embedded .res ..       54.0 Degree
PLAT969_ALERT_5_G The 'Henn et al.' R-Factor-gap value ..........      41.138 Note
              Predicted wR2: Based on SigI**2  0.20 or SHELX Weight  7.56
PLAT978_ALERT_2_G Number C-C Bonds with Positive Residual Density.          0 Info
PLAT992_ALERT_5_G Repd & Actual _reflns_number_gt Values Differ by          3 Check
```

---

0 **ALERT level A** = Most likely a serious problem - resolve or explain

```
 0 ALERT level B = A potentially serious problem, consider carefully
 5 ALERT level C = Check. Ensure it is not caused by an omission or oversight
25 ALERT level G = General information/check it is not something unexpected

10 ALERT type 1 CIF construction/syntax error, inconsistent or missing data
 2 ALERT type 2 Indicator that the structure model may be wrong or deficient
 5 ALERT type 3 Indicator that the structure quality may be low
 5 ALERT type 4 Improvement, methodology, query or suggestion
 8 ALERT type 5 Informative message, check
```

It is advisable to attempt to resolve as many as possible of the alerts in all categories. Often the minor alerts point to easily fixed oversights, errors and omissions in your CIF or refinement strategy, so attention to these fine details can be worthwhile. In order to resolve some of the more serious problems it may be necessary to carry out additional measurements or structure refinements. However, the purpose of your study may justify the reported deviations and the more serious of these should normally be commented upon in the discussion or experimental section of a paper or in the "special_details" fields of the CIF. checkCIF was carefully designed to identify outliers and unusual parameters, but every test has its limitations and alerts that are not important in a particular case may appear. Conversely, the absence of alerts does not guarantee there are no aspects of the results needing attention. It is up to the individual to critically assess their own results and, if necessary, seek expert advice.

Publication of your CIF in IUCr journals

A basic structural check has been run on your CIF. These basic checks will be run on all CIFs submitted for publication in IUCr journals (*Acta Crystallographica*, *Journal of Applied Crystallography*, *Journal of Synchrotron Radiation*); however, if you intend to submit to *Acta Crystallographica Section C* or *E* or *IUCrData*, you should make sure that full publication checks are run on the final version of your CIF prior to submission.

Publication of your CIF in other journals

Please refer to the *Notes for Authors* of the relevant journal for any special instructions relating to CIF submission.

PLATON version of 04/06/2025; check.def file version of 30/05/2025

Datablock I - ellipsoid plot

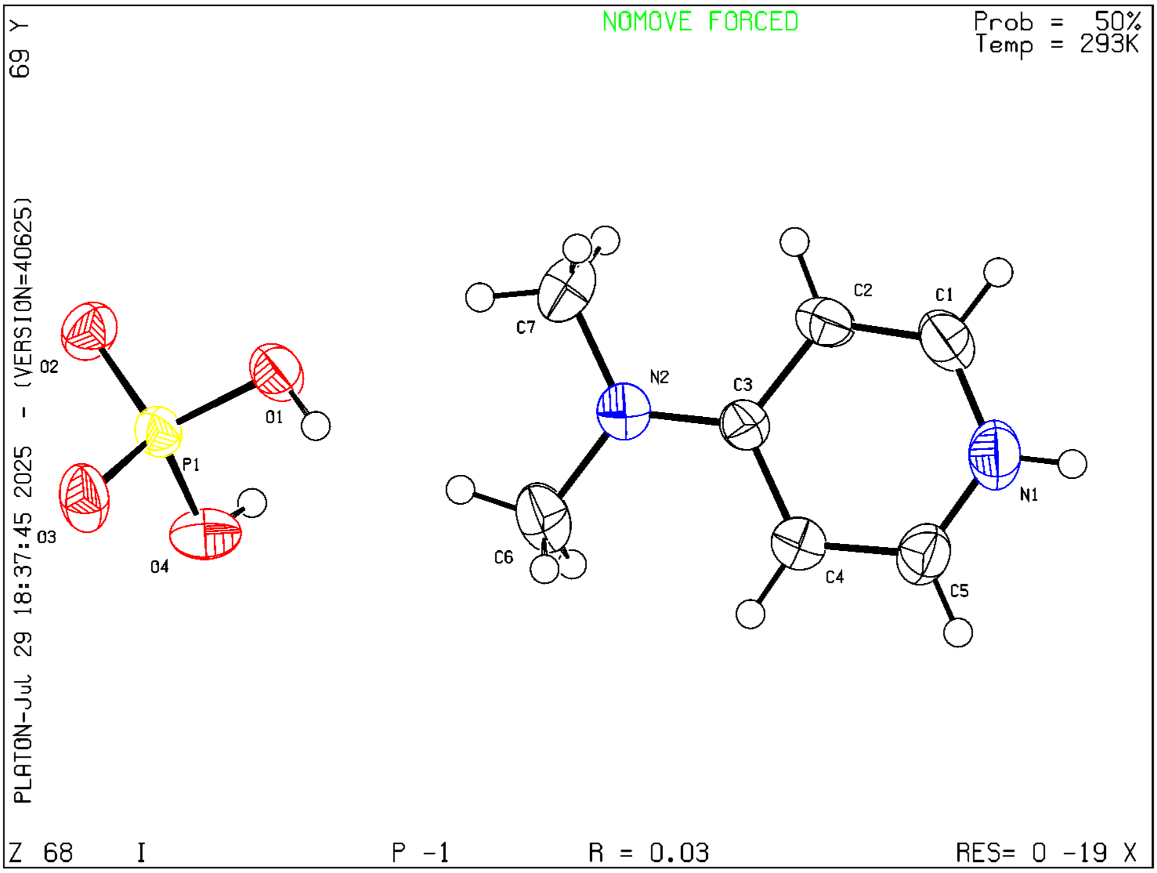

Supplement: Supplementary file 1 [file DataSheet1.pdf]
